# Transcriptomic HIV-1 reservoir profiling reveals a role for mitochondrial functionality in HIV-1 latency

**DOI:** 10.1371/journal.ppat.1012822

**Published:** 2025-01-10

**Authors:** Shirley Man, Jade Jansen, Stefanie Kroeze, Teunis B. H. Geijtenbeek, Neeltje A. Kootstra

**Affiliations:** 1 Department of Experimental Immunology, Amsterdam UMC Location University of Amsterdam, Amsterdam, Netherlands; 2 Amsterdam institute for Immunology and Infectious Diseases, Amsterdam, Netherlands; Vaccine Research Center, UNITED STATES OF AMERICA

## Abstract

Identifying cellular and molecular mechanisms maintaining HIV-1 latency in the viral reservoir is crucial for devising effective cure strategies. Here we developed an innovative flow cytometry-fluorescent *in situ* hybridization (flow-FISH) approach for direct *ex vivo* reservoir detection without the need for reactivation using a combination of probes detecting abortive and elongated HIV-1 transcripts. Our flow-FISH assay distinguished between HIV-1-infected CD4+ T cells expressing abortive or elongated HIV-1 transcripts in PBMC from untreated and ART-treated PWH from the Amsterdam Cohort Studies. This flow-FISH method was employed to isolate CD4+ T cells expressing abortive or elongated HIV-1 transcripts from five ART-naïve PWH for transcriptomic analysis by 3’ RNA sequencing. Supervised cluster analysis identified several differentially expressed mitochondrial genes in infected CD4+ T cells with abortive HIV-1 transcripts compared to cells containing elongated HIV-1 transcripts. Notably, enhancing mitochondrial function induced HIV-1 transcription in PBMC from PWH. Our data strongly suggests that cellular metabolism is involved in maintaining HIV-1 latency and show that improving mitochondrial functions induces HIV-1 transcriptional activity in PWH. These findings underline the relevance of metabolic regulation in HIV-1 infection, and support the development of strategies modulating immunometabolism to target viral latency.

## Author summary

Our limited understanding of HIV-1 latency remains a major obstacle in developing effective cure strategies. To better characterize latently infected cells, we developed a flow-FISH assay that is able to distinguish between HIV-1-infected cells expressing either abortive or elongated HIV-1 transcripts without the need for cellular activation. Transcriptomic profiling of these distinct cell populations from peripheral blood mononuclear cells (PBMCs) of people with HIV-1 (PWH) identified several differentially expressed genes that were related to the mitochondrial machinery and its functions. Notably, *ex vivo* stimulation of mitochondrial activity increased HIV-1 transcription in PBMC from PWH. Thus, our study underscores the role of cellular metabolism and mitochondrial function in HIV-1 latency.

## Introduction

A major hurdle to HIV-1 cure is the persistence of the viral reservoir, a population of immune cells that harbors latent copies of the integrated HIV-1 genome. The lack of viral antigen expression associated with viral latency hampers detection and subsequent elimination of these infected cells by the immune system. Thus, these latently infected cells are a sanctuary for HIV-1 and are a source of viral rebound upon antiretroviral therapy (ART) interruption [1].

A proper understanding of the HIV-1 reservoir is crucial for the development of cure strategies aiming to effectively target and eliminate infected cells. HIV-1 can be found in all CD4+ T cell subsets, in which particularly the central memory (TCM), effector memory (TEM) and transitional memory T cells (TTM) have been identified as major reservoirs [2–5]. However, other HIV-1-enriched subsets have also been reported, such as regulatory T cells (Tregs), T follicular helper cells (Tfh), T helper 1 (Th1), and Th17 cells [6–9]. Antibody-based phenotyping studies have shown that cells enriched for HIV-1 express an array of surface proteins, including, but not limited to, immune checkpoint markers (PD-1, TIGIT, LAG-3), and activation and proliferation markers (CD25, HLA-DR, Ki67) [10–12]. More recently, Sun et al. analyzed the phenotype of HIV-1-infected cells and found an ensemble of surface proteins that mostly confer protection against immune-mediated killing, suggesting that their differing phenotypic signatures are likely to arise from immune selection within the compartmentalized microenvironment [13]. This heterogeneity of the infected cell pool has been pointed out by several other recent studies on HIV-1 persistence, where HIV-1 was found to be residing in CD4+ T cell subsets with varying phenotypes, driven by different cellular programs and mechanisms [14–17]. The heterogeneous composition and variable phenotype of the viral reservoir underline its complexity and emphasize the need for further characterization.

At the molecular level, HIV-1 latency is characterized by perturbed viral transcription due to for instance inefficient transcriptional elongation, chromatin organization and epigenetic modifications [18]. Inefficient transcriptional elongation mostly occurs in the absence of the viral transcriptional activator protein Tat, leading to the production of mostly short abortive transcripts [19–22]. These abortive transcripts lack polyadenylation and comprise the transcription initiation site and the trans-activation response (TAR) region of the HIV-1 genome, a highly conserved RNA sequence that is located at the 5’ end of every viral RNA transcript. Latently infected cells and cells containing a transcriptionally active HIV-1 genome, can thus be distinguished by the presence of abortive transcripts and/or elongated HIV-1 RNAs. At present, the cellular conditions that induce or maintain viral latency are still relatively unknown.

Here, we have aimed to characterize the transcriptomic profiles of the HIV-1 reservoir cells that contain either abortive or elongated HIV-1 transcripts in order to elucidate cellular mechanisms maintaining viral latency. Standard methods employed in reservoir studies rely on *ex vivo* reactivation of HIV-1, which changes the phenotypic landscape of latently infected cells, rendering obtained results different from their *in vivo* state. We have therefore developed an assay using flow cytometry to detect HIV-1 transcripts with branched DNA technology and fluorescent *in situ* hybridization (flow-FISH) that allowed for *ex vivo* isolation of HIV-1-infected cells containing either abortive or elongated HIV-1 transcripts from peripheral blood mononuclear cells (PBMCs) from people with HIV-1 (PWH), without the need for reactivation. Gene profiling analysis of these isolated cells strongly suggests an association between mitochondrial functioning and transcriptional activity of the viral reservoir, and supports the development of strategies modulating immunometabolism to target viral latency.

## Results

### Detection of HIV-1-infected CD4+ T cells expressing abortive or elongated HIV-1 transcripts in PWH

**Fig 1:**
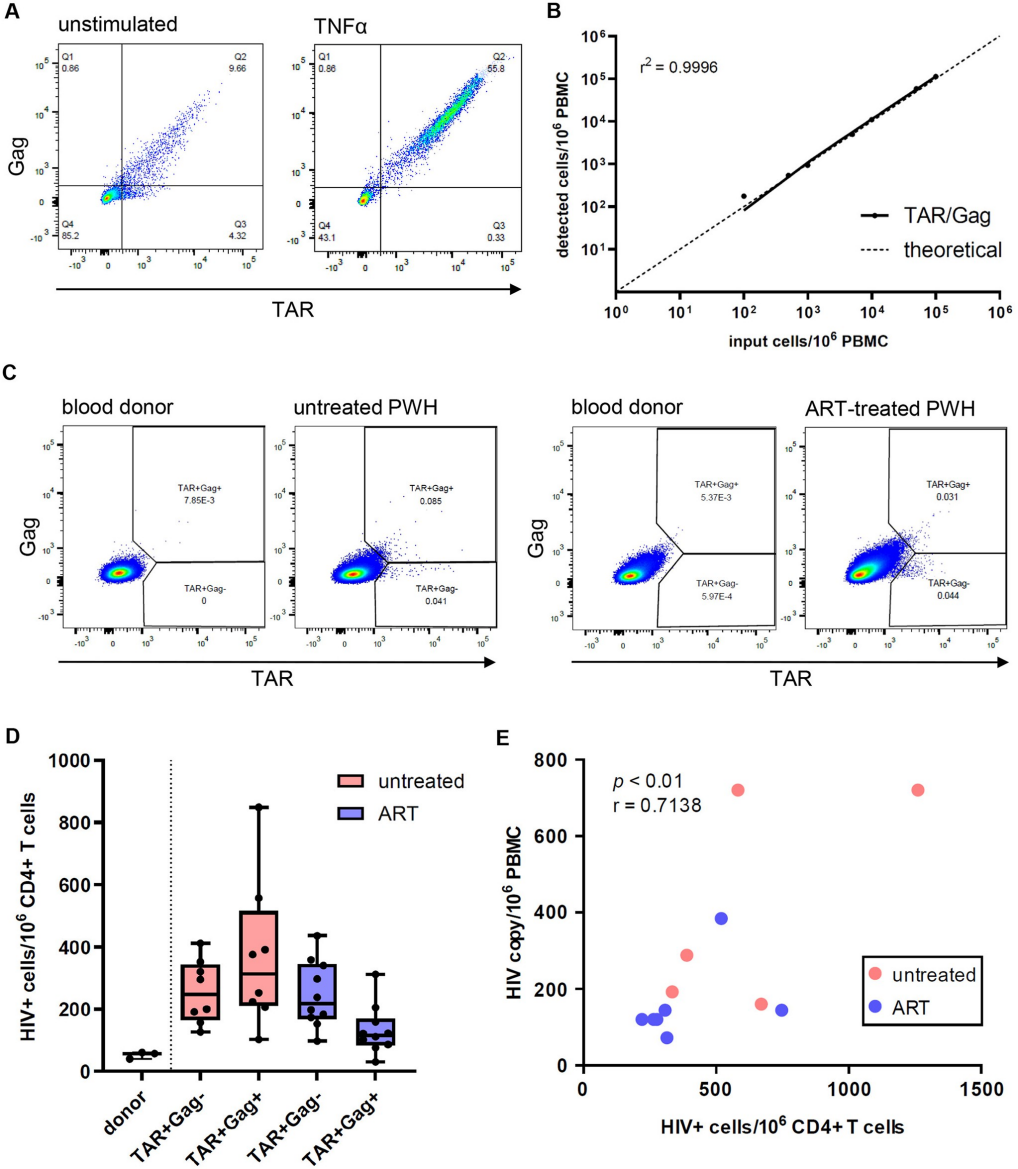
Flow-FISH detection of HIV-1-infected cells. (A) Flow cytometric quadrant plots showing TAR+Gag+ (Q2) and TAR+Gag- (Q3) cells in unstimulated and TNFα-activated ACH-2. (B) Numbers of detected TNFα-activated ACH-2 cells by flow-FISH assay (i.e. TAR+Gag+ and TAR+Gag-) spiked at different ratios in donor PBMC. The theoretical curve is represented by the dashed line. Simple linear regression was performed (r^2^ = 0.9996). (C) Representative flow cytometric plots showing HIV-1-infected cells containing elongated HIV-1 transcripts (TAR+Gag+) and abortive HIV-1 transcripts (TAR+Gag-) in the CD4+ T cell population of PBMCs from an untreated and ART-treated individual with HIV-1. Blood donors from independent experiments are shown as negative control. (D) Total number of cells containing abortive (TAR+Gag-) or elongated (TAR+Gag+) HIV-1 transcripts per 10^6^ CD4+ T cells detected by flow-FISH in PBMCs from untreated (N = 8) and ART-treated PWH (N = 10). Blood donors (N = 3) are included as control. (E) Graph showing the correlation between HIV-1 *pol* PCR (HIV copy/10^6^ PBMC) and flow-FISH assay (HIV+ cells/10^6^ CD4+ T cells). Non-parametric Spearman correlation was performed (r = 0.7138, *p* < 0.01).

For the detection of HIV-1-infected cells, we designed a flow-FISH assay in which flow cytometry is combined with branched DNA technology using fluorescent probes that amplify the TAR region, which is potentially present in all HIV-1 mRNAs including abortive RNAs, and probes that amplify the Gag region for the detection of elongated HIV-1 mRNAs (S1 Table). This combination of probes allows for flow cytometric quantification of HIV-1-infected cells expressing only abortive TAR RNA and HIV-1-infected cells expressing elongated HIV-1 transcripts. The assay was validated using the ACH-2 latency cell line in which low level viral transcriptional activity was observed prior to stimulation (Fig 1A). Following stimulation with TNF-α, a 5-fold increase in TAR+Gag+ cells was observed while the TAR+Gag- population was no longer detected. These data strongly suggest that the TAR/Gag flow-FISH assay distinguishes cells with either abortive or elongated HIV-1 transcription. Moreover, we spiked TNF-α-stimulated ACH-2 at different ratios into PBMCs from blood donors and observed strong linearity of the flow-FISH assay (Fig 1B).

Next, *ex vivo* PBMC from 8 untreated participants with a chronic HIV-1 infection and 10 ART-treated participants (characteristics displayed in Table 1) were analyzed by TAR/Gag flow-FISH. In addition, PBMC from blood donors were included to control for background fluorescence and non-specific binding of the probes. We detected CD4+ T cells expressing either abortive (TAR+Gag-) or elongated (TAR+Gag+) HIV-1 transcripts in PWH (Fig 1C). The number of infected CD4+ T cells differed between untreated and ART-treated PWH, ranging from 303–1261 and 220–747 infected cells/10^6^ CD4+ T cells, respectively (Fig 1D). Importantly, our flow-FISH probes distinguished between HIV-1-infected cells expressing only abortive TAR RNA or HIV-1-infected cells expressing elongated transcripts, and the number of infected CD4+ T cells with only abortive transcripts (range 127-412/10^6^) was substantially lower than the number of CD4+ T cells with elongated transcripts (range 103-892/10^6^) in PBMCs from untreated PWH. Although the frequencies of infected CD4+ T cells with abortive transcripts were similar for both untreated and ART-treated PWH (range 98-436/10^6^), the fraction of CD4+ T cells containing elongated transcripts was smaller in PBMC from PWH on ART (range 30-312/10^6^). An association was observed between the number of HIV-1 positive cells determined by HIV-1 *pol* PCR and the number of HIV-1 positive cells detected by flow-FISH (Fig 1E; r = 0.7138; p<0.01). Our data therefore strongly suggest that the TAR/Gag flow-FISH assay allows for *ex vivo* detection of infected CD4+ T cells expressing either abortive or elongated HIV-1 transcripts in PBMC from PWH without the need for any pre-stimulation.

**Table 1:** Participant characteristics.

Characteristic	Value		
Total participants	25		
Gender: male	100%		
	**Flow cytometry quantification**		**Flow cytometry sorting**
	Untreated	Treated	Untreated
Number	8	10	7
Age (median years, IQR)	34 (28–42)	39 (35–49)	28 (27–39)
Absolute CD4 count (median x10E^9^ cells/L, IQR)	0.69 (0.56–0.77)	0.61 (0.54–0.65)	0.52 (0.39–0.65)
Absolute CD8 count (median x10E^9^ cells/L, IQR)	0.94 (0.75–1.11)	1.04 (0.93–1.37)	1.35 (1.08–1.4)

### Transcriptional profiling of HIV-1-infected CD4+ T cells from PWH

**Fig 2:**
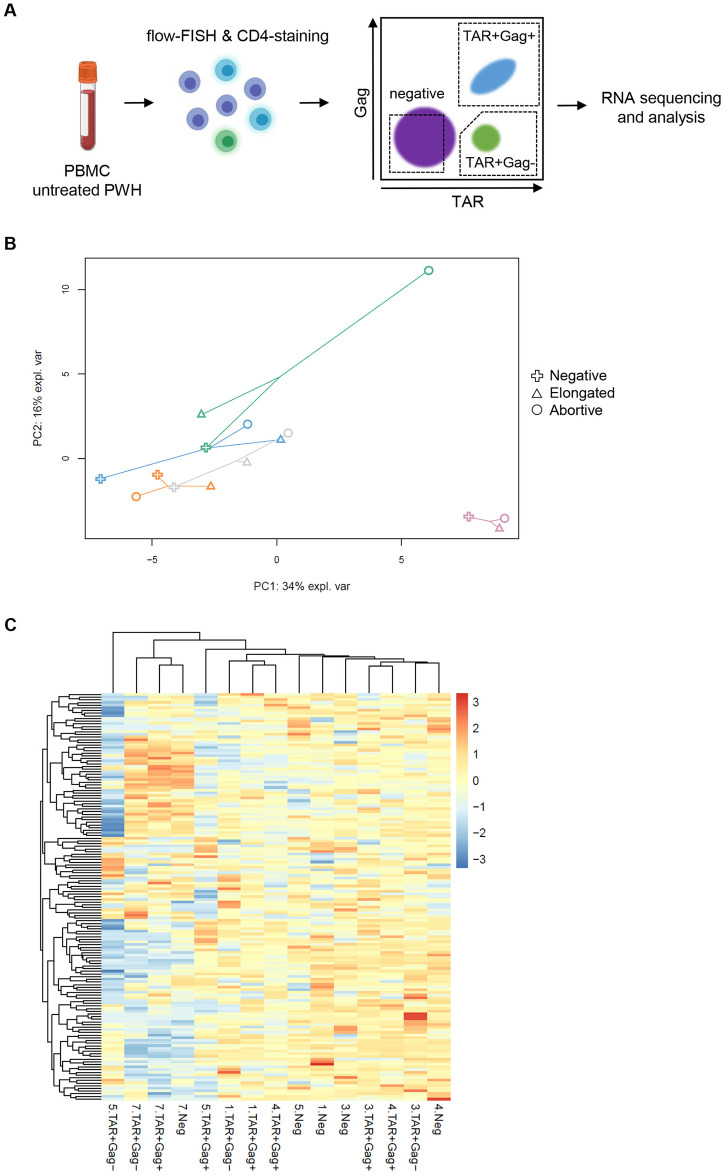
Unsupervised analysis of transcriptomes of isolated HIV-1-infected cells. (A) Schematic overview of the cell isolation procedure (created using BioRender.com). Flow-FISH was used for the sorting of HIV-1-infected (i.e. elongated HIV-1 transcripts; TAR+Gag+ or abortive HIV-1 transcripts; TAR+Gag-), and probe-negative (negative) CD4+ T cells from PBMCs of PWH (N = 5). (B) PCA plot showing clustering of samples. Each individual is represented by a different color, and cell populations are indicated by different symbols. Samples from the same individuals are connected by lines. (C) Heatmap plot showing the clustering of samples (horizontal axis) by gene expression (vertical axis). Colors represent log2-transformed counts per million (CPM) values.

To gain a better understanding of the transcriptional profile of cells expressing abortive or elongated HIV-1 transcripts, we sorted CD4+ T cells that contained either abortive HIV-1 transcripts (TAR+Gag-) or elongated HIV-1 transcripts (TAR+Gag+), and probe-negative CD4+ T cells (negative) from PBMC of 7 PWH (characteristics displayed in Table 1; Fig 2A; S2 Table). RNAseq samples were selected based on their library size, leading to the exclusion of samples from two individuals (<100,000 total number of reads) and resulting in a total set of 15 samples from 5 PWH for further analyses. Unsupervised principal component analysis (PCA) showed modest clustering of samples by cellular fraction (Fig 2B). The gene expression of the cellular fractions was found to be heterogeneous, whereas expression patterns of several samples derived from the same individuals were seen to be similar (Fig 2C). This observed transcriptomic similarity of CD4+ T cell fractions within individuals suggests that supervised clustering might be more appropriate for the identification of differences between cellular fractions.

**Fig 3:**
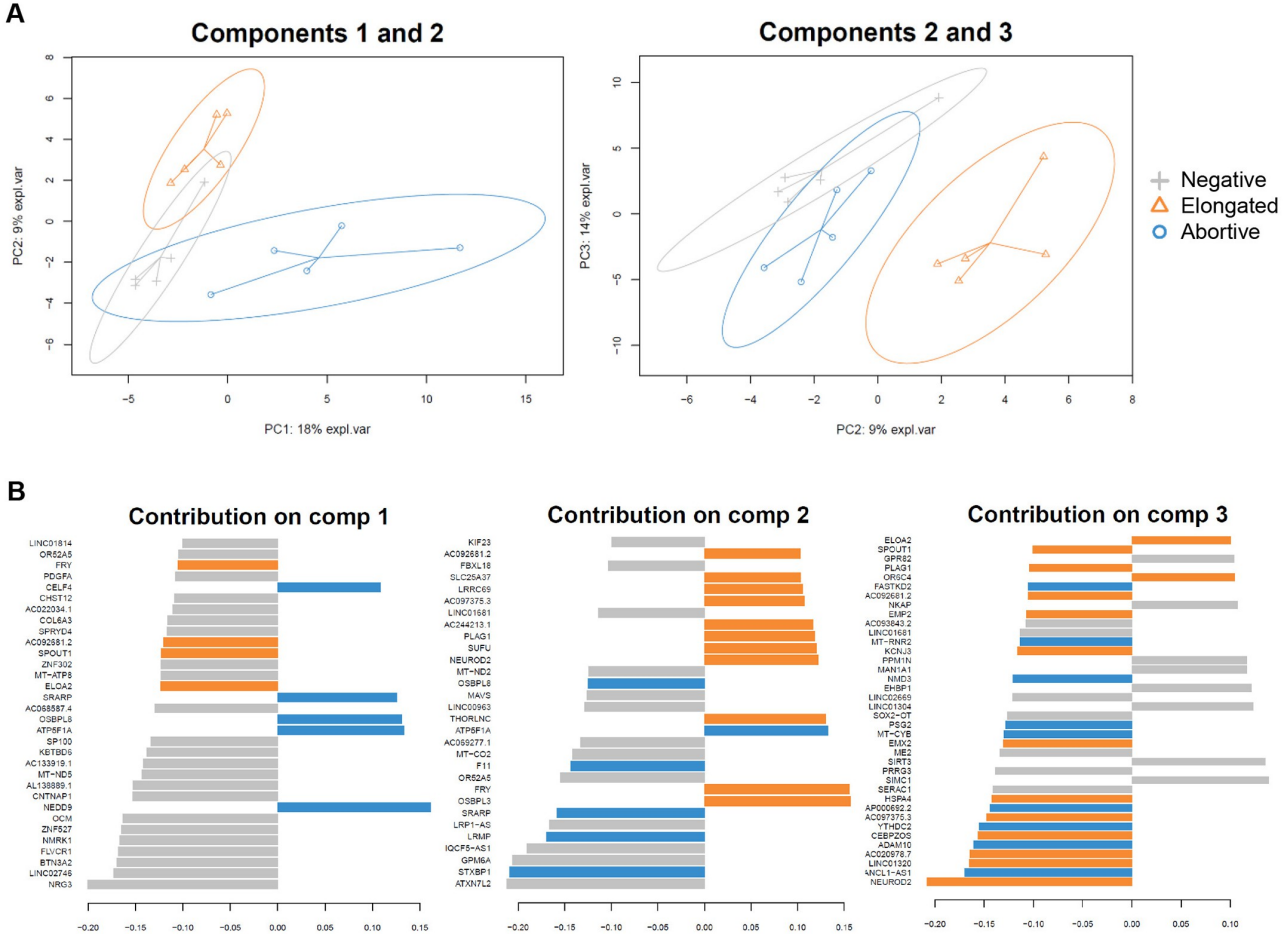
Supervised PLS-DA of HIV-1-infected CD4+ T cells. (A) PLS-DA score plots showing separation of the isolated populations along three components, with centroids and ellipses depicting the median coordinates and 95% confidence interval (CI), respectively. (B) Loading plots listing the top contribution genes of each PLS-DA component (loading cutoff ≤-0.1 and ≥0.1), with importance ranking from bottom to top. Colors indicate the population in which the gene has the highest expression.

Partial least squares discriminant analysis (PLS-DA) was used to identify a gene expression profile that allows for separation of probe-negative CD4+ T cells, and infected CD4+ T cells with abortive or elongated HIV-1 transcripts. Projected components 1, 2, and 3 separated the different cell fractions and accounted for gene expression variance of 18%, 9%, and 14% respectively (Fig 3A). Component 1 and 2 contributed to separation of the CD4+ T cells with abortive transcripts (left panel), whereas components 2 and 3 contributed to additional separation between the population of cells that contained elongated transcripts or cells that were probe-negative (right panel). The top contributing genes of the PLS-DA components (loading cutoff ≤-0.1 and ≥0.1) were extracted to assess the gene expression profiles of the three populations (Fig 3B; S3 Table). The top contributing genes for component 1 were mostly differentially expressed in the probe negative fraction, while components 2 and 3 were defined by genes expressed in all three cell populations. This analysis shows that infected cells with abortive or elongated HIV-1 transcripts, and HIV-1 transcript-negative cells from PWH can be distinguished by a unique transcriptomic profile.

### Differentially expressed genes between HIV-1-infected cells expressing abortive or elongated HIV-1 transcripts

**Fig 4:**
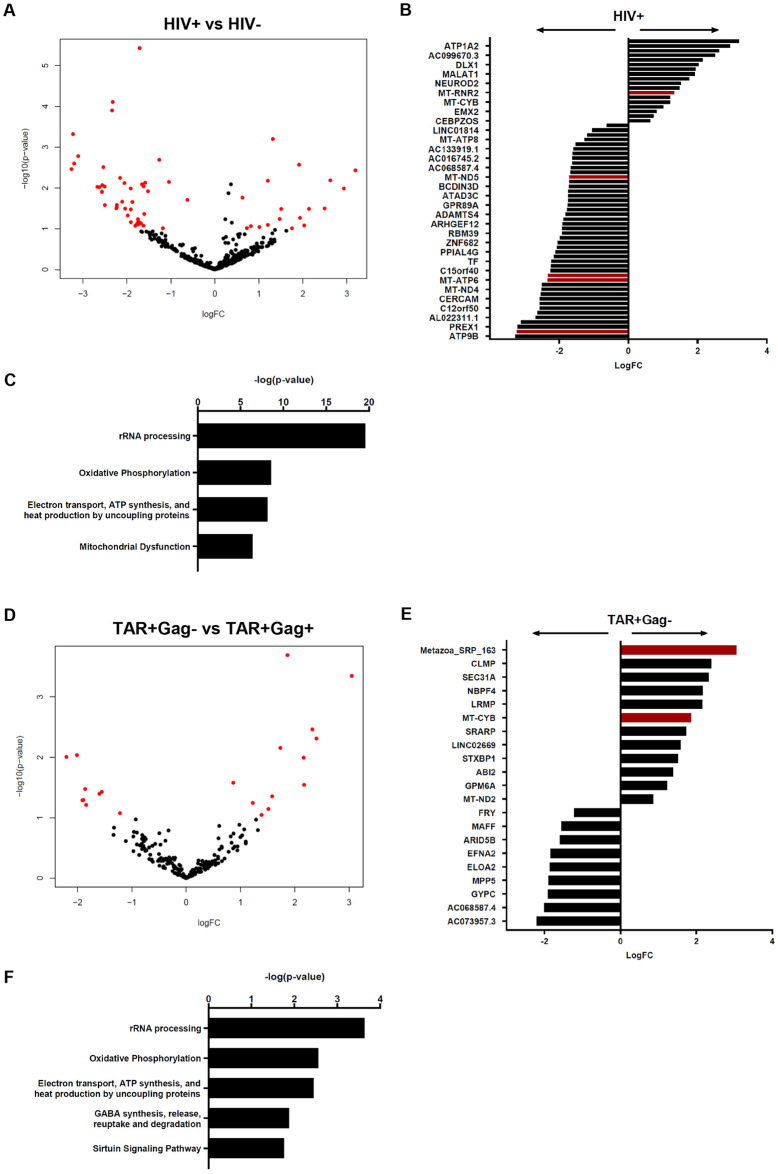
Supervised DGE analysis of HIV-1-infected CD4+ T cells. (A) Volcano plot of paired DGE analysis for HIV+ compared to HIV-with DEGs shown in red (logFC ≤-0.5 and ≥0.5 and nominal *p* <0.1). (B) Barplot showing up- and downregulation of DEGs for HIV+ compared to HIV-, with red bars denoting DEGs with adjusted *p* <0.05. (C) Horizontal barplot showing the top canonical pathways for DEGs found in HIV+ compared to HIV-. (D) Volcano plot of paired DGE analysis for the population with abortive HIV-1 transcripts compared to the population with elongated HIV-1 transcripts with DEGs shown in red (logFC ≤-0.5 and ≥0.5 and nominal *p* <0.1). (E) Barplot showing up- and downregulation of DEGs for the population with abortive HIV-1 transcripts compared to the population with elongated HIV-1 transcripts, with red bars denoting DEGs with adjusted *p* < 0.05.

Having found specific gene profiles for each population, we next performed a supervised differential gene expression (DGE) analysis to identify singular genes that could be used as biomarkers for HIV-1-infected cells. To specify genes for infected cells (HIV+), TAR+Gag- and TAR+Gag+ CD4+ T cell populations were grouped together for comparison to the probe-negative population (HIV-). DGE analysis for paired samples identified 64 differentially expressed genes (DEGs) with a fold change ≤-0.5 and ≥0.5 and nominal p <0.1, of which 18 were up- and 46 were downregulated in the HIV+ population compared to the HIV- population (Fig 4A and 4B; S4 Table). 5 DEGs differed significantly (adjusted p <0.05) between HIV+ and HIV- cells: *MT-ND5*, *MT-ATP6*, *MT-ND4L*, *MT-RNR2*, and non-coding (lnc)RNA *ADAMTS9-AS2*. Mitochondrial 16S ribosomal RNA-encoding *MT-RNR2* was upregulated in the HIV+ CD4+ T cell population, whereas the other mitochondrial genes, encoding for subunits of respiratory complexes I and V, were downregulated. “rRNA processing”, “Oxidative Phosphorylation”, “Electron transport, ATP synthesis, and heat production by uncoupling proteins”, and “Mitochondrial Dysfunction” were among the top enriched canonical pathways as determined by Ingenuity Pathway Analysis (IPA; Fig 4C).

Additionally, to identify genes that distinguish cells with abortive HIV-1 transcripts from cells with elongated HIV-1 transcripts, we analyzed the gene expression of these two cell populations (Fig 4D and 4E). Paired samples comparison showed 21 DEGs (fold change ≤-0.5 and ≥0.5 and nominal p <0.1), of which 12 were up- and 9 were downregulated in cells containing abortive HIV-1 transcripts. Two of these DEGs were found to be significantly upregulated (adjusted p <0.05): *MT-CYB*, which encodes for the mitochondrial respiratory complex III subunit cytochrome b, and signal recognition particle RNA *Metazoa SRP 163*, which is suggested to be part of ribonucleoprotein complexes. The top enriched canonical pathways for these genes were “rRNA processing”, “Oxidative Phosphorylation”, “Electron transport, ATP synthesis, and heat production by uncoupling proteins”, “GABA synthesis, release, reuptake and degradation”, and “Sirtuin Signaling Pathway” (Fig 4F). Our analysis identified genes that were differentially expressed in HIV-1-infected compared to uninfected CD4+ T cells, and found DEGs that were more specific for infected CD4+ T cells containing either abortive or elongated HIV-1 transcripts. Notably, the majority of these genes were found to be involved in the mitochondrial machinery and related processes.

### Mitochondrial activation of PBMCs from PWH increases HIV-1 transcriptional activity

**Fig 5:**
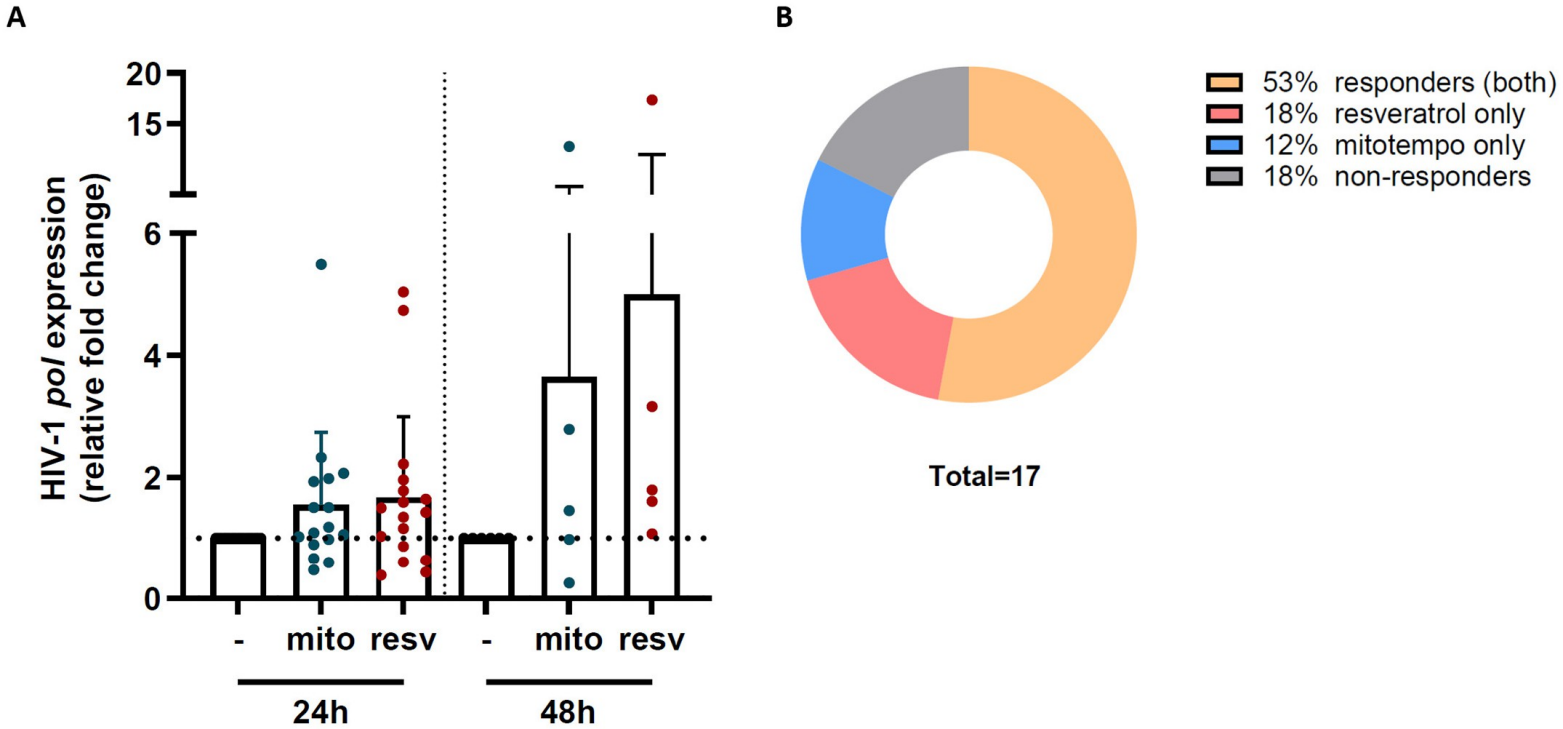
HIV-1 *pol* gene expression in PBMC of PWH upon antioxidant stimulation. (A) Barplot showing the gene expression of HIV-1 *pol* in PBMCs of PWH following 24 hour (N = 17) or 48 hour (N = 5) treatment with MitoTempo (10 μM) or trans-resveratrol (10 μM), relative to the untreated condition. (B) Pie chart depicting the number of individuals with increased HIV-1 *pol* expression upon 24 hour treatment to both stimuli (responders), MitoTempo only, trans-resveratrol only, or without increased expression (non-responders).

As we identified several DEGs involved in mitochondrial processes, we investigated whether induction of mitochondrial metabolism reactivates HIV-1 transcription in latently infected cells of PWH. We treated PBMCs from PWH (patient characteristics S5 Table) with antioxidant compounds MitoTempo or trans-resveratrol for 24 and 48 hours (N = 17 and 5, respectively), and determined HIV-1 transcriptional activity by quantitative *pol* PCR. Notably, increased HIV-1 transcription upon mitochondrial activation for 24 and 48 hours was observed in PBMC from the majority of individuals (Fig 5A). We observed an induction of HIV-1 transcription in 53% of the individuals in response to both stimuli, and 12% and 18% in response to only MitoTempo or trans-resveratrol, respectively (Fig 5B). Thus, our data suggest that HIV-1 transcriptional activity in infected cells from PWH is dependent on the mitochondrial activity and that mitochondrial stimulation leads to HIV-1 reactivation.

## Discussion

Insights into the characteristics of the HIV-1 reservoir and mechanisms of viral latency are critical for the development of effective cure strategies. Here, we developed a flow-FISH assay using probes specifically targeting abortive and elongated HIV-1 RNAs to isolate HIV-1-infected CD4+ T cells from PBMCs of PWH without the need for reactivation. Transcriptomic analysis of these isolated cells was performed to determine their gene expression profiles and uncover underlying processes involved in HIV-1 transcription. Importantly, we identified several genes related to the mitochondrial machinery that differed between HIV-1-infected CD4+ T cells containing either abortive or elongated HIV-1 transcripts, and showed that enhancing mitochondrial activity was able to reactivate HIV-1 in PBMC from the majority of untreated PWH.

Significant efforts are ongoing to develop techniques for accurate detection of the HIV-1 reservoir, however, thus far no optimal method has been developed yet. Our approach and probe design adds to established flow-FISH detection methods [23–26], where the targeted combination of the Gag sequence serves as proxy for transcriptional elongation, and the TAR sequence as marker for viral latency in the absence of elongated HIV-1 mRNAs. Abortive TAR transcripts are suggested to be a by-product of ineffective and incomplete transcription of the HIV-1 genome, and have been identified in latency cell lines and in resting CD4+ T cells from ART-treated PWH [21,22,27]. However, the presence of abortive TAR RNAs does not dictate whether the HIV-1 provirus is intact or whether it harbors deleterious mutations, hence our probe combination does not distinguish between cells harboring replication-competent or defective proviruses. Moreover, it is also possible that transcriptionally active cells harbor provirus with large deletions in Gag, which will not be detected by the Gag probe and as such will be misidentified as latently infected cells by the flow-FISH assay. By using a probe that spans 1.5 kb of the Gag sequence we expect these chances to be slim, though this possibility cannot be entirely excluded. With the flow-FISH assay we detected between 220–1261 HIV-1 RNA+ cells/10^6^ CD4+ T cells for untreated and ART-treated PWH, which correlated with proviral DNA levels as determined by PCR. Previously reported median numbers of HIV-1-infected cells in the peripheral blood of untreated viremic PWH ranged from 82–473 cells/10^6^ CD4+ T cells, as quantified by methods such as flow-FISH- or PCR-based viral RNA detection [23,25,28]. The varied number of HIV-1+ cells detected with our assay could be due to differences in participants and probe selection.

Unsupervised cluster analysis of CD4+ T cells expressing only TAR, both TAR and Gag transcripts, or cells that had no detectable levels of HIV-1 RNA showed modest clustering of samples based on cell population, but mostly indicated clustering by the individual participant. Similar observations were recently made by Clark et al. following a whole-transcriptome clustering analysis on sorted HIV-DNA+ cells, where they noted sample clustering by participant and significant transcriptomic variation among HIV-DNA+ cell samples [14]. The lack of clustering of the HIV-1-infected cells obtained from the different participants may be due to differences in the cellular composition of the HIV-1 positive cell population as HIV-1 can be present in different CD4+ T (memory) cells, such as activated/quiescent cells or T helper subsets (Th1/Th2/Th17/Tfh). However, by performing a PLS-DA that separates the cell populations in a supervised manner we were able to determine profiles of gene expression defining the isolated populations containing either abortive or elongated HIV-1 transcripts.

We identified several genes encoding for components of the mitochondrial respiratory complexes to be differentially expressed in HIV-1-infected cells, as well as in cells containing only abortive HIV-1 transcripts. Moreover, pathway analysis indicated that the DEGs of the HIV+ population were mainly related to cellular metabolism as defined by the top pathways i.e. electron transport, oxidative phosphorylation, and mitochondrial dysfunction. HIV-1 has been shown to selectively infect highly metabolic CD4+ T cells, independently of T cell activation status [29]. Moreover, enhanced metabolic activity has been suggested to be important for HIV-1 infection as infection increases oxidative phosphorylation and glycolysis in primary CD4+ T cells, whereas oxidative phosphorylation stimulates virus replication [30]. In another study, glycolysis was found to be downregulated in latently infected cells and restoration of this metabolic pathway was shown upon viral reactivation [31]. In line with these studies, our identified gene expression profiles that highly suggest that the mitochondrial functionality and the metabolic state of the cell are important for HIV-1 transcriptional activation and viral latency. Indeed, treatment of PBMC from PWH with antioxidant compounds known to improve mitochondrial function, such as readily-available sirtuin-1 activator resveratrol [32], increased production of elongated HIV-1 *pol* transcripts within 24 hours. It should be noted that resveratrol is a polyphenol that not only stimulates mitochondrial biogenesis and abrogates mitochondrial dysfunction [33,34], but also regulates other processes such as apoptosis, inflammation, and proliferation [35], which in their turn may also influence HIV-1 transcription. However, resveratrol has been shown to inhibit NF-kB signaling [36,37] and therefore it is unlikely that the observed latency reversal (Fig 5) is due to a direct effect of resveratrol on cellular activation or HIV-1 transcription. Other studies have also shown that resveratrol can reactivate latent HIV-1 through increasing histone acetylation [38], activation of P-TEFb [39], and Tat accumulation via AKT/FOXO1 signaling [40]. As we observed similar effects with both resveratrol and MitoTempo, a mitochondria-targeted antioxidant that can reverse mitochondrial dysfunction [41,42], our data suggests that their common antioxidant activity is causing the increased HIV-1 transcription. Transcriptional activation of HIV-1 using these compounds was not observed in all PWH, which is most likely due to the heterogeneity of the viral reservoir and different mechanisms of viral latency as previously observed by others [43]. However, our data suggest that mitochondrial stimulation and metabolic processes are important regulators of HIV-1 transcriptional activity and are involved in viral reactivation.

Earlier profiling studies have reported an enrichment of HIV-1 in CD4+ T cells positive for co-inhibitory receptors like PD-1, CTLA-4, TIGIT and LAG-3, which is indicative of an exhausted and dysfunctional phenotype [10]. However, we did not find genes coding for these co-inhibitory receptors to be specifically upregulated in our HIV+ cell populations. The expression of these co-inhibitory receptors is most likely not induced by HIV-1 infection itself, but rather are expressed on cells that are more susceptible to infection.

Recent single-cell characterization studies have concluded that the viral reservoir is highly heterogeneous, and attempts have been made to group HIV-1-infected cells by gene and protein expression. Accordingly, HIV-1-infected cells were found to display a signature of cell survival and resistance to immune-mediated killing [13,14], and more recently have been grouped in antiviral, inflammatory, or cytotoxic clusters [17]. Although single-cell transcriptomics generally allows for high resolution RNA transcript identification, these platforms usually capture only a limited number of HIV-1-infected cells for analysis. We therefore chose to perform RNAseq using flow-FISH-sorted cell populations, which allows for the selection of HIV-1-infected cells that contain either abortive or elongated HIV-1 transcripts and therefore will likely identify gene expression profiles associated with these distinct populations. However, our employed method requires fixation of the cells, and only allowed for a limited sequencing depth, thus transcripts expressed at lower levels in the total cell population were likely not detected. Nevertheless, our bulk sequencing approach did reveal an overall gene signature, pointing to an aberrant expression of genes related to metabolic regulation and RNA processing in HIV-1-infected cells, as well as infected cells expressing only abortive HIV-1 transcripts. It is known that metabolic processes are closely coupled to the cellular state, and that homeostasis of differentiated cytotoxic T cells require a different energy demand than naïve or resting T cells (as reviewed in [44–46]). We also observed the differential expression of lncRNAs—a category of underexplored transcripts that are suggested to play diverse cellular roles, e.g. at the level of chromatin remodeling and transcriptional regulation, and which have been found to regulate T cell functions [47,48]. Our identified DEGs and putative cellular processes may thus underlie these clustering observations made by others.

Our TAR/Gag flow-FISH approach and transcriptomic dataset contribute to the growing understanding of the HIV-1 reservoir. Our findings support the hypothesis that cells harboring HIV-1 have an altered metabolic profile compared to uninfected cells, and suggest that lower mitochondrial activity maintains latency in HIV-1-infected cells. This study highlights the role of immunometabolism in HIV-1 infection and latency, and warrant the therapeutic targeting or reversal of HIV-1 latency through metabolic modulation.

## Methods

### Ethics statement

The ACS has been conducted in accordance with the ethical principles set out in the declaration of Helsinki. Written informed consent was provided by all participants. Authors had no access to information that could identify individual participants during or after data collection. The study was approved by the institutional medical ethical committee of the Academic Medical Center (2007–182) and the ethics advisory body of the Sanquin Blood Supply Foundation in Amsterdam, The Netherlands.

### Study participants

PBMC samples from participants from the Amsterdam Cohort Studies (ACS) with an HIV-1 infection [49] were selected for this study. All participants were seropositive for HIV-1 at entry into the study, and either had an untreated course of infection or initiated ART during the chronic phase of infection and have been stably on suppressive ART for at least 6 months with an undetectable viral load. PBMC samples from healthy blood donors were obtained through the Dutch national blood bank (Sanquin) in Amsterdam, The Netherlands.

### TAR/Gag flow-FISH assay

For the flow-FISH assay, a set of probes specific for HIV-1 RNA transcripts containing TAR and/or Gag were designed based on the sequences display in S1 Table (Affymetrix, Santa Clara, CA, USA). These probes were used to target and amplify TAR for the detection of abortive and elongated HIV-1 transcripts, and Gag for the detection of elongated HIV-1 transcripts. The Primeflow RNA Assay (Invitrogen, Waltham, MA, USA) was used for the flow-FISH detection of HIV-1-infected cells in PBMC from PWH according to manufacturer’s instructions, with some modifications. Briefly, PBMC (5–10×10^6^) were first stained with fluorescently labelled CD4-PE antibody (RPA-T4 clone; Biolegend, San Diego, CA, USA) for 30 min at 4°C. To preserve short abortive transcripts, cells were incubated with Primeflow microRNA Pretreatment Buffer (Invitrogen) for 15 min at RT in the dark. Cells were then fixed and permeabilized, followed by an additional intracellular fixation step. Next, cells were incubated with flow-FISH probes (S1 Table) for 2 hours at 40°C during a hybridization step. Subsequently, cells were subjected to two branched-DNA amplification steps (1.5 and 2 hours, respectively) at 40°C followed by an incubation (1 hour at 40°C) with fluorescent probes that bind the branched-DNA amplifiers. Fluorescence was measured using the FACSCanto II device (BD Biosciences, Franklin Lakes, NJ, USA) or the FACSAria device (BD Biosciences) for cell sorting. Data was analyzed by FlowJo software version 10 (TreeStar, Ashland, OR, USA). Validation was performed using unstimulated and TNFα- (10 ug/ul, PeproTech, Cranbury, NJ, USA) activated ACH-2 cells (NIH AIDS Reagent Program; RRID:CVCL_0138), and by spiking these cells at different ratios in donor PBMC. In each separate experiment, PBMC from blood donors and TNF-α-stimulated ACH-2 cells were included as negative and positive assay controls, respectively.

### Cellular HIV load

DNA from PBMC was isolated using the AllPrep RNA/DNA kit (Qiagen, Venlo, The Netherlands) and DNA concentrations were determined by Nanodrop. The cell-associated HIV load was determined by PCR following a single genome amplification (SGA). In short, a minimum of 10 PCR reactions were performed using GoTaq polymerase containing 2.5–250 ng DNA. Primer sets used were as follows: for primary PCR, HIV Pol-F 5′- TTAGTCAGTGCTGGAATCAGG-3′ and Pol-D 5′-GCTACATGAACTGCTACCAGG-3′ were used; and for nested PCR, HIV Pol-B 5′-TAACCTGCCACCTGTAGTAGCAAAAGAAAT-3′ and Pol-E 5′-ATGTGTACAATCTAGTTGCCA-3′. The PCR cycles for both primary and nested PCR reactions were as follows; denaturation 5 min at 94°C followed by 35 cycles of 15 s at 94°C, 30 s at 50°C, 45 s at 72°C and finally followed by 1 cycle of 5 min at 72°C. PCR reactions were visualized on 1% agarose gel. When at least two thirds of reactions were positive, it was presumed that each reaction contained no more than one proviral DNA copy. The proviral HIV-DNA copy number per cell was calculated assuming a concentration of 6 pg DNA per cell.

### RNA sequencing and data processing

Following aforementioned flow-FISH staining, cells from PBMC of PWH were sorted in 100-cell aliquots by the FACSAria device and resuspended in Cell Lysis Buffer (1.25 μl/sample, QIAseq UPX 3’ Transcriptome Kit, Qiagen, Hilden, Germany), RNase Inhibitor (0.3125 μl/sample, QIAseq UPX 3’ Transcriptome Kit), and proteinase K (0.1 μg/sample; Merck, Rahway, NJ, USA) for 30 min at 56°C, followed by 15 min inactivation at 95°C. Samples were transferred to 96-well single-use Cell ID RT Plates (QIAseq UPX 3’ Transcriptome Kit), and cDNA libraries were prepared according to the Qiaseq 3’ Transcriptome Kit manual. DNA concentration of the samples was measured with a Qubit Fluorometer (Invitrogen). The quality of the libraries was confirmed by capillary electrophoresis (Agilent DNA 7500 Chip; Agilent, Santa Clara, CA, USA), and the libraries were quantified using qPCR. The libraries were then sequenced on a NextSeq 500 sequencing instrument according to the manufacturer’s instructions’ (100 bp read length for read 1 and 27 bp for read 2). The bcl2fastq Conversion Software (Illumina, San Diego, CA, USA) was used to demultiplex raw data and generate FASTQ files. Raw sequencing reads were demultiplexed using the "Demultiplex QIAseq UPX 3’ reads" tool of the CLC Genomics Workbench (version 12.0.4; Qiagen), and demultiplexed sequencing reads were processed using the "Quantify QIAseq UPX 3’ workflow" with default settings. The reads were then mapped to the Human genome GRCh38 and annotated using the NCBI RefSeq GRCh38.p11 gene annotation. Samples with a low number of reads (<100,000) were omitted from downstream transcriptomic analysis, leading to the exclusion of 2 participants. Mapped gene counts from samples belonging to the same cell population and participant were pooled together. Lowly expressed genes were filtered, and libraries were normalized using the EdgeR package [50] in RStudio (v.4.1.0). The dataset generated in this study has been deposited at https://www.ncbi.nlm.nih.gov/geo/ with the accession number GSE260588.

### RNA sequencing data analysis

RStudio was used for the analysis of the sequencing data. Principal component analysis (PCA) was performed using EdgeR, and visualization was carried out with the ggplot2 package [51]. Partial Least Squares-Discriminant Analysis (PLS-DA) was performed using the mixOmics package (version 6.3.2) [52]. Differential gene expression (DGE) analysis was performed using EdgeR. Genes with a logFC of ≥0.5 and p-value <0.1 were considered differentially expressed genes (DEGs). Where indicated p-values were corrected for multiple testing using the Benjamini-Hochberg procedure. Pathway overrepresentation was performed with Ingenuity Pathway Analysis (2023 Winter Release, Qiagen).

### Mitochondrial stimulation

PBMCs of PWH were stimulated for 24 or 48 hours with or without antioxidant compounds MitoTempo (10 μM; Saint Louis, MO, Sigma-Aldrich) and trans-resveratrol (10 μM; Sigma-Aldrich) at a concentration of 2 × 10^6^ cells/ml in Iscove’s modified Dulbecco medium (IMDM) supplemented with 10% FCS, penicillin (100 U/ml), streptomycin (100 U/ml), and IL-2 (20 U/ml).

### RNA isolation and RT-qPCR

RNA from PBMCs was isolated using the RNeasy Plus Mini Kit (Qiagen) according to the manufacturer’s instructions. cDNA was synthesized with random hexamer primers using the M-MLV Reverse Transcriptase Kit (Promega, Madison, WI, USA), according to the protocol of the manufacturer. RT-qPCR reactions were performed using the GoTaq qPCR system with SYBR Green (Promega) in a 7500 Fast Real-Time PCR System (Applied Biosystems, Waltham, MA, USA). Primer sequences used were: b-actin (Fw) 5′-GGGTCAGAAGGATTCCTATG-3′ and (Rv) 5′-GGTCTCAAACATGATCTGGG-3′, and HIV-pol-B (Fw) 5′-TAACCTGCCACCTGTAGTAGCAAAAGAAAT-3′ and pol-E (Rv) 5′-ATGTGTACAATCTAGTTGCCA-3′. Amplification conditions consisted of a pre-incubation stage at 94°C for 3 min, and an amplification stage consisting of 3 steps at 94°C for 20 sec, 59°C for 30 sec, and 72°C for 30 sec (45 cycles). A melting curve analysis was performed to confirm purity of the PCR product and primer specificity. Gene expression was normalized using the 2^-ΔΔCt method.

### Statistical analysis

Graphpad Prism (version 9.3.1; GraphPad Software, San Diego, CA, USA) was used for data analysis and data visualization. Flow-FISH assay linearity was determined by simple linear regression. Correlations between reservoir measurements were determined using non-parametric Spearman correlation analysis. A p-value < 0.05 was considered statistically significant.

## Supporting information

S1 Table Sequences used for design of FISH probes. (PDF)

S2 Table Frequencies of CD4+ T cells with abortive or elongated HIV transcripts in PBMC of PWH. (PDF)

S3 Table PLS-DA top variables of populations (from components 1, 2, and 3). (PDF)

S4 Table List of DEGs and functions. (PDF)

S5 Table Participant characteristics. (PDF)

S1 Fig Gating strategy of flow-FISH. Representative FACS plots showing the gating strategy in PBMC of a blood donor.
(PDF)
