# Supplementary material for: Transcriptomic HIV-1 reservoir profiling reveals a role for mitochondrial functionality in HIV-1 latency
Source: PLoS Pathog. 2025 Jan 10;21(1):e1012822. doi: 10.1371/journal.ppat.1012822 (PMC11723532; doi:10.1371/journal.ppat.1012822)
Supplement: S1 Table — (PDF) [file ppat.1012822.s001.pdf]

S1 Table. Sequences used for design of FISH probes.

	Target sequence (from HXB2 reference genome)
TAR	gggtctctctggttagaccagatctgagcctgggagctctctggctaactagggaacccac
TAR+5’LTR	ggtctctctggttagaccagatctgagcctgggagctctctggctaactagggaacccactgcttaagcctcaataaagc<br>ttgccttgagtgcttcaagtagtgtgtgcccgtctgttgtgtgactctggtaactagagatccctcagacccttttagtcag<br>tgtggaaaatctctagcagtggcgcccgaacagggacctgaaagcgaaagggaaaccagaggagctctctcgacgca<br>ggactcggcttgctgaagcgcgcacggcaagaggcgaggggcggcgactggtgagtacgccaaaaattttgactagc<br>ggaggctagaaggagagag
Gag	atgggtgcgagagcgtcagtattaagcgggggagaattagatcgatgggaaaaaattcggttaaggccagggggaaa<br>gaaaaaatataaattaaaacatatagtatgggcaagcagggagctagaacgattcgcagttaatcctggcctgttaga<br>aacatcagaaggctgtagacaaatactgggacagctacaaccatcccttcagacaggatcagaagaacttagatcatt<br>atataatacagtagcaaccctctattgtgtgcatcaaaggatagagataaaagacaccaaggaagctttagacaagat<br>agaggaagagcaaaacaaaagtaagaaaaaagcacagcaagcagcagctgacacaggacacagcaatcaggtca<br>gccaaaattaccctatagtgcagaacatccaggggcaaatggtacatcaggccatatcacctagaactttaaatgcatg<br>ggtaaaagtagtagaagagaaggctttcagcccagaagtgatacccatgttttcagcattatcagaaggagccacccc<br>acaagatttaaacaccatgctaaacacagtggggggacatcaagcagccatgcaaatgttaaaagagaccatcaatg<br>aggaagctgcagaatgggatagagtgcatccagtgcatgcagggcctattgcaccaggccagatgagagaaccaagg<br>ggaagtgacatagcaggaactactagtacccttcaggaacaaataggatggatgacaaataatccacctatcccagta<br>ggagaaatttataaaagatggataatcctgggattaaataaaatagtaagaatgtatagccctaccagcattctggac<br>ataagacaaggaccaaaggaaccctttagagactatgtagaccggttctataaaactctaagagccgagcaagcttca<br>caggaggtaaaaaattggatgacagaaaccttgttggtccaaaatgcgaacccagattgtaagactattttaaaagca<br>ttgggaccagcggctacactagaagaaatgatgacagcatgtcagggagtaggaggacccggccataaggcaagagt<br>tttggctgaagcaatgagccaagtaacaaattcagctaccataatgatgcagagaggcaattttaggaaccaaagaaa<br>gattgttaagtgtttcaattgtggcaaagaagggcacacagccagaaattgcagggcccctaggaaaaagggctgttg<br>gaaatgtggaaaggaaggacaccaaatgaaagattgtactgagagacaggctaattttttagggaagatctggccttc<br>ctacaagggaaggccagggaattttcttcagagcagaccagagccaacagccccaccagaagagagcttcaggtctg<br>gggtagagacaacaactccccctcagaagcaggagccgatagacaaggaactgtatcctttaacttccctcaggtcac<br>tctttggcaacgacccctcgtcacaataa
