# Supplementary material for: Transcriptomic HIV-1 reservoir profiling reveals a role for mitochondrial functionality in HIV-1 latency
Source: PLoS Pathog. 2025 Jan 10;21(1):e1012822. doi: 10.1371/journal.ppat.1012822 (PMC11723532; doi:10.1371/journal.ppat.1012822)
Supplement: S2 Table — (PDF) [file ppat.1012822.s002.pdf]

**S2 Table. Frequencies of CD4+ T cells with abortive or elongated HIV-1 transcripts in PBMC of PWH.**

| Participant | TAR+Gag+ | TAR+Gag- | probe-negative |
|---|---|---|---|
| 1 | 1,660 | 842 | 100,000 |
| 2 | 8,070 | 758 | 100,000 |
| 3 | 19,607 | 2,634 | 100,000 |
| 4 | 50,996 | 1,003 | 100,000 |
| 5 | 3,562 | 209 | 100,000 |
| 6 | 6,176 | 257 | 100,000 |
| 7 | 5,127 | 224 | 100,000 |
