# Supplementary material for: Transcriptomic HIV-1 reservoir profiling reveals a role for mitochondrial functionality in HIV-1 latency
Source: PLoS Pathog. 2025 Jan 10;21(1):e1012822. doi: 10.1371/journal.ppat.1012822 (PMC11723532; doi:10.1371/journal.ppat.1012822)
Supplement: S3 Table — (PDF) [file ppat.1012822.s003.pdf]

S3 Table. PLS-DA top variables of populations (from components 1, 2, and 3).

Abortive	Elongated	Negative
NEDD9	CEBPZOS	NRG3
ATP5F1A	LINC01320	LINC02746
OSBPL8	SPOUT1	BTN3A2
SRARP	LRRC69	FLVCR1
CELF4	SUFU	NMRK1
STXBP1	FRY	ZNF527
LRMP	EMX2	OCM
F11	OR6C4	CNTNAP1
LANCL1-AS1	HSPA4	AL138889.1
ADAM10	ELOA2	MT-ND5
YTHDC2	SLC25A37	AC133919.1
AP000692.2	EMP2	KBTBD6
MT-CYB	AC092681.2	SP100
PSG2	OSBPL3	AC068587.4
NMD3	THORLNC	MT-ATP8
MT-RNR2	AC020978.7	ZNF302
FASTKD2	AC097375.3	SPRYD4
	PLAG1	COL6A3
	KCNJ3	AC022034.1
	NEUROD2	CHST12
	AC244213.1	PDGFA
	CEBPZOS	OR52A5
		LINC01814
		ATXN7L2
		GPM6A
		IQCF5-AS1
		LRP1-AS
		MT-CO2
		AC069277.1
		LINC00963
		MAVS
		MT-ND2
		LINC01681
		FBXL18
		KIF23
		SERAC1
		SIMC1
		PRRG3
		SIRT3
		ME2
		SOX2-OT
		LINC01304
		LINC02669
		EHBP1
		MAN1A1

	PPM1N
	AC093843.2
	NKAP
	GPR82
