# Supplementary material for: Transcriptomic HIV-1 reservoir profiling reveals a role for mitochondrial functionality in HIV-1 latency
Source: PLoS Pathog. 2025 Jan 10;21(1):e1012822. doi: 10.1371/journal.ppat.1012822 (PMC11723532; doi:10.1371/journal.ppat.1012822)
Supplement: S4 Table — (PDF) [file ppat.1012822.s004.pdf]

S4 Table. List of DEGs and functions.

Gene	Identifier (Ensembl)	Gene product function or related process	HIV-1 transcriptional activity
MT-CYB	ENSG00000198727	cellular respiration; electron transport coupled proton transport	latent
Metazoa SRP 163	ENSG00000277721	ribonucleoprotein complex (putative)	latent
SEC31A	ENSG00000138674	vesicle budding from the endoplasmic reticulum (ER)	latent
CLMP	ENSG00000166250	type I transmembrane protein	latent
SRARP	ENSG00000183888	intracellular estrogen receptor signaling pathway	latent
LRMP/IRAG2	ENSG00000118308	immune system process, vesicle fusion; vesicle targeting	latent
MT-ND2	ENSG00000198763	aerobic respiration, mitochondrial electron transport	latent
NBPF4/NBPF6	ENSG00000196427	member of the neuroblastoma breakpoint family (NBPF)	latent
LINC02669	ENSG00000233321	RNA regulation (putative)	latent
GPM6A	ENSG00000150625	calcium ion transmembrane transport	latent
STXBP1	ENSG00000136854	SNARE signaling pathway, integration of energy metabolism	latent
ABI2	ENSG00000138443	cytoskeleton organization, RAC Signaling	latent
AC068587.4	ENSG00000283674	RNA regulation (putative)	active
AC073957.3	ENSG00000273151	RNA regulation (putative)	active
ELOA2	ENSG00000206181	transcription elongation	active
MAFF	ENSG00000185022	transcription regulator	active
ARID5B	ENSG00000150347	transcription regulator	active
MPP5/ PALS1	ENSG00000072415	MAGUK gene superfamily	active
GYPC	ENSG00000136732	integral membrane glycoprotein	active
EFNA2	ENSG00000099617	cell-cell signaling	active
FRY	ENSG00000073910	cell morphogenesis	active
MT-RNR2	ENSG00000210082	ribosome function	latent
MALAT1	ENSG00000251562	ribonucleoprotein complex, transcriptional regulator (putative)	latent
WASF2	ENSG00000158195	cell migration, adhesion, and cell morphology	active
AC073957.3	ENSG00000273151	RNA regulation (putative)	active
ATP1A2	ENSG00000018625	ATP binding, sodium/potassium-transport	active
CEBPZOS	ENSG00000218739	ncRNA located in the vicinity of CEBPZ; gene expression modulation	active
AC099670.3	ENSG00000285525	RNA regulation (putative)	latent

NEUROD2	ENSG00000171532	member of NeuroD family of bHLH transcription factors	active
MT-RNR1	ENSG00000211459	ribosome function	latent
NEDD9	ENSG00000111859	signal transduction, cell adhesion and migration	latent
LINC01320	ENSG00000228262	RNA regulation (putative)	latent
DLX1	ENSG00000144355	transcription regulator	latent
EMX2	ENSG00000170370	transcription regulator	active
LANCL1-AS1	ENSG00000234281	RNA regulation (putative)	latent
AC020978.7	ENSG00000263276	RNA regulation (putative)	active
PSMD3	ENSG00000108344	subunit of the 26S proteasome, ATPase activity, protein deubiquitination	active
