# Supplementary material for: Transcriptomic HIV-1 reservoir profiling reveals a role for mitochondrial functionality in HIV-1 latency
Source: PLoS Pathog. 2025 Jan 10;21(1):e1012822. doi: 10.1371/journal.ppat.1012822 (PMC11723532; doi:10.1371/journal.ppat.1012822)
Supplement: S5 Table — (PDF) [file ppat.1012822.s005.pdf]

S5 Table. Participant characteristics.

Characteristic	Value
Total participants	17
Gender: male	100%
Age (median years, IQR)	38 (30-45)
Absolute CD4 count (median x10E^{9} cells/L, IQR)	0.46 (0.20-0.66)
Absolute CD8 count (median x10E^{9} cells/L, IQR)	0.80 (0.60-1.20)
