# Supplementary material for: Transcriptomic HIV-1 reservoir profiling reveals a role for mitochondrial functionality in HIV-1 latency
Source: PLoS Pathog. 2025 Jan 10;21(1):e1012822. doi: 10.1371/journal.ppat.1012822 (PMC11723532; doi:10.1371/journal.ppat.1012822)
Supplement: S1 Fig — Representative FACS plots showing the gating strategy in PBMC of a blood donor. (PDF) [file ppat.1012822.s006.pdf]

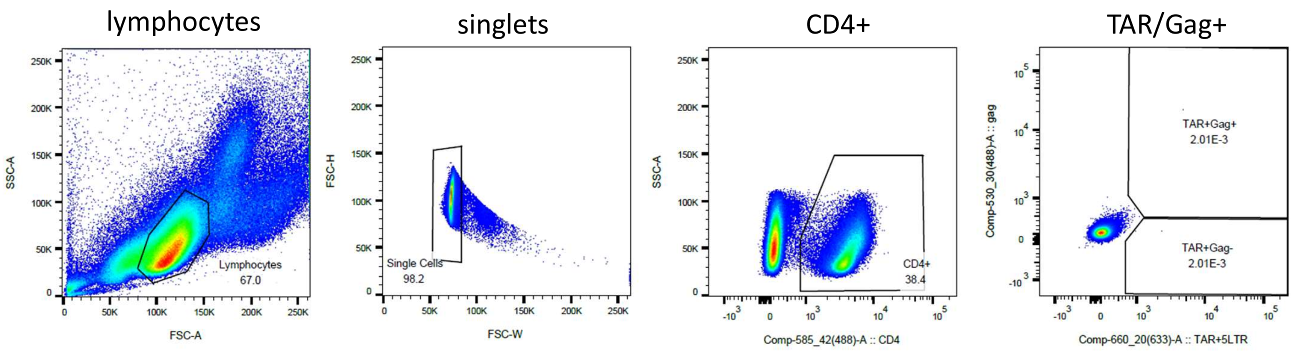

S1 Figure. Gating strategy of flow-FISH. Representative FACS plots showing the gating strategy in PBMC of a blood donor.
